# Rapid and simple analysis of amphetamine-type illegal drugs using excitation–emission matrix fluorescence coupled with parallel factor analysis

**DOI:** 10.1080/20961790.2017.1349600

**Published:** 2017-07-24

**Authors:** Buyi Xu, Yi Ye, Linchuan Liao

**Affiliations:** a Sichuan Provincial Department of Public Security, Chengdu, China; b Department of Forensic Toxicological Analysis, West China School of Preclinical and Forensic Medicine, Sichuan University, Chengdu, China

**Keywords:** Forensic science, illegal drugs, excitation–emission matrix fluorescence, PARAFAC

## Abstract

Nowadays, the abuse of illegal drugs has been an increasingly grim problem in the world. Excitation–emission matrix fluorescence combined with parallel factor analysis was used to make a quantitative analysis of the simulated amphetamine-type illegal drugs. Satisfactory results were achieved for simultaneous determination of methamphetamine (MAM) and 3, 4-methylenedioxymethamphetamine (MDMA) in the presence of adulterants. The average recoveries were (99.8 ± 0.6)% and (101.6 ± 5.7)% for MAM and MDMA, respectively. Figures of merit including root-mean-square error of calibration and prediction, sensitivity and selectivity were investigated to evaluate the performance of the proposed method. The limits of detection were 0.054 and 0.002 1 μg/mL for MAM and MDMA, respectively.

## Introduction

According to the recently available World Drug Report 2016, the number of illegal drug users has risen rapidly over the last five years. It is estimated that 250 million people, corresponding to 10% of the world population, had used an illicit drug in the previous year (http://www.unodc.org/wdr2016). Amphetamine-type stimulants (ATS) such as methamphetamine (MAM) and 3, 4-methylenedioxymethamphetamine (MDMA), two of the most common ATS known as “ice” and “ecstasy”, are widely abused among young people. These illegal drugs are potent in stimulating the central nervous system, reducing fatigue and inducing euphoria. Continuous use of those illegal drugs will make a person produce psychological dependence.

There are many adulterants in the seized illegal drugs. For example, some seized MDMA tablets contained MAM, MDMA, amphetamine (AM), ephedrine, ketamine, cocaine (COC) or caffeine (CAF) [1]. The adulterants in the tablets may enhance the stimulant effects of drug mixture, disguise the existence of illicit drugs or use for secret substitution of a more expensive illicit drug with a cheaper substance. Therefore, a more rapid method for simultaneous determination of ATS in a mixture with adulterants would be highly desirable for efficient battle against illegal drug trafficking.

Due to its unambiguous identification of compounds and good sensitivity, chromatography is a powerful method for determination of ATS in biological samples, e.g. gas chromatographic system equipped with a flame ionization detector (GC-FID) for analysis of MAM and related compounds in urine [2], high performance liquid chromatography with fluorescence detection for the quantification of MDMA and MDA in hair samples [3], liquid chromatography mass spectrometry/mass spectrometry (LC-MS/MS) method for the determination of ATS in urine and hair [4–6], thin-layer chromatography coupled with matrix-assisted laser desorption/ionization mass spectrometry for the determination of MDMA and its main metabolites in urine and organs [7]. In addition, capillary electrophoresis-laser induced fluorescence was also used for the detection of AM derivatives [8]. For bulk detection of suspicious street illegal samples, precise analyses were typically performed using GC-MS or LC-MS in the laboratory [9]. Takahashi et al. [10] created a psychoactive drug data library to identify the illegal drugs in purchased products. Global seizures of ATS have risen dramatically over the last decade. It has been reported that 54 tons of ATS were seized worldwide in 2003, while it increased to approximately 170 tons in 2016 (http://www.unodc.org/wdr2016). To support a large number of illegal drug measurements, a rapid and simple method is required for identification of seized illegal drugs instead of time-consuming chromatographic method.

**Figure 1.**
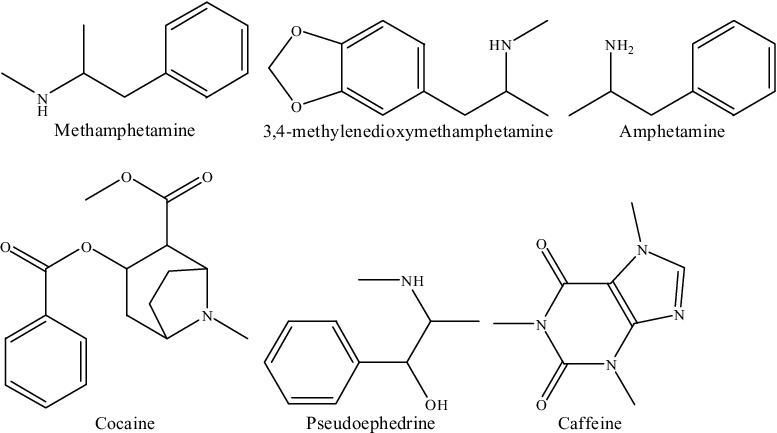
The structures of methamphetamine (MAM), 3, 4-methylenedioxymethamphetamine (MDMA) and their adulterants.

One alternative to chromatographic methods is fluorescence spectroscopy. It is possible to identify the ATS illegal drugs based on fluorescence due to their aromatic structures (Figure 1). Excitation–emission matrix (EEM) fluorescence was proposed for monitoring these illegal drugs in seized street samples [11,12]. Mazina et al. [13] proposed a novelty method for detection of illegal drugs (COC, heroin and MDMA) in seized street samples using EEM combined with multilayer perceptron artificial neural networks. However, these proposed fluorescence methods were just qualitative analyses of these illicit drugs, in which only “detected/or not detected” response was obtained. Modern drug laws required that a seized suspicious street sample be characterized for both the illegal substances present and the quantity of each of those substances. The fluorescence method is simple and rapid; however, due to the existence of the adulterants in ATS mixtures, it is impossible to quantify the illegal drugs only using the fluorescence method due to its featureless.

Generally, chemometric algorithms, such as parallel factor analysis (PARAFAC), were proposed to decompose the EEM complex fluorescence signal into individual fluorescence spectra. EEM combined with chemometric algorithm has been successfully applied for the study of polycyclic aromatic hydrocarbons and pesticides in natural water [14], methylcoumarin in cosmetics [15], organic pollutants in environmental analysis [16], aromatic amino acids in plasma and urine [17,18], and the adulterants in adulterant-brandy blends [19,20]. Therefore, EEM coupled with PARAFAC algorithm could provide a new avenue for rapidly determining the illegal drugs with adulterants.

The objectives of this study are: (1) to characterize the fluorescence signatures of several ATS illegal drugs; (2) to develop an innovative method for bulk detection of ATS illicit drugs in a mixture with adulterants; and (3) to evaluate the performance of the PARAFAC algorithm using statistic parameters.

## Materials and methods

### Regents and solutions

The standards of COC hydrochloride, AM hydrosulphate, CAF, pseudoephedrine (PSE) hydrochloride, MAM hydrochloride and MDMA hydrochloride were supplied by the Ministry of Public Security of the People's Republic of China.

Stock solutions of ATS including AM, MAM, MDMA, COC and PSE were prepared in deionized water at the concentration of 100 mg/L, and CAF was at a concentration of 500 mg/L due to their low fluorescence intensity. The working standard solutions were prepared by appropriate dilution of the stock solution with deionized water. All the stock solutions and the working solutions were stored at 4 °C until analysis.

The simulated ATS street samples were prepared by adding appropriate volumes of adulterants to pure MAM and MDMA solutions. The concentrations of MAM and MDMA with their adulterants are listed in Table 1. In the present study, we only considered one adulterant mixed in the pure MAM or MDMA. For multi adulterants presented in the illegal drugs, it will be discussed in our future work. Additionally, due to the similarity of the structure between MAM and its adulterants (AM and PSE), it cannot be resolved from those mixtures. Thus, we do not discuss the above two adulterants in the following text.

**Table 1.** Concentrations of MAM and MDMA in the simulated mixtures (mg/L).

	Mixture 1			Mixture 2			Mixture 3			Mixture 4		
Samples	No.	MAM	MDMA	No.	MAM	COC	No.	MAM	CAF	No.	MDMA	COC
Calibration samples	1^#^	1.0	0	11^#^	1.0	1.0	21^#^	1.0	100	31^#^	0.01	1.0
	2^#^	2.0	0.09	12^#^	3.0	0.8	22^#^	2.0	90	32^#^	0.02	0.9
	3^#^	4.0	0.07	13^#^	4.0	0.7	23^#^	3.0	80	33^#^	0.04	0.7
	4^#^	5.0	0.06	14^#^	6.0	0.5	24^#^	5.0	60	34^#^	0.06	0.5
	5^#^	6.0	0.05	15^#^	7.0	0.4	25^#^	7.0	40	35^#^	0.07	0.4
	6^#^	8.0	0.03	16^#^	9.0	0.2	26^#^	9.0	20	36^#^	0.09	0.2
	7^#^	10.0	0.01	17^#^	10.0	0.1	27^#^	10.0	10	37^#^	0.1	0.1
Prediction samples	8^#^	3.0	0.08	18^#^	2.0	0.9	28^#^	4.0	70	38^#^	0.03	0.8
	9^#^	7.0	0.04	19^#^	5.0	0.6	29^#^	6.0	50	39^#^	0.05	0.6
	10^#^	9.0	0.02	20^#^	8.0	0.3	30^#^	8.0	30	40^#^	0.08	0.3

### EEM fluorescence measurements

Fluorescence measurements were performed on an F-7000 fluorescence spectrofluorometer (Hitachi, Japan). All measurements were recorded using a 10 mm quartz cell at room temperature. The EEMs were collected at the excitation wavelengths (λ_ex_) between 230 and 350 nm, and emission wavelengths (λ_em_) between 250 and 550 nm with an interval of 2 nm. Both the excitation and emission slits were set at 5 nm, and the scan speed was 12 000 nm/min. Additionally, deionized water was regularly recorded during the sample measurements. No fluorescence signal of the interested materials or other interferences was found in the deionized water (Figure S1).

### The three-way tri-linear model

EEM measurements can provide a data matrix, and a series of data matrices obtained for multiple samples enable to make up a data cube **X**. The PARAFAC algorithm will decompose the data cube **X** into *A*, *B* and *C* loading matrices, which can be expressed as follows:

(1) $$x_{ijk}=\sum_{n=1}^{N}a_{in}b_{jn}c_{kn}+e_{ijk},\;\;i=1,\ldots ,I;\;j=1,\ldots ,J;\;k=1,\ldots ,K\;$$

where *x_ijk_* is an element of **X**; it represents the fluorescence intensity of sample number *k* at the excitation wavelength number *i* with emission wavelength number *j*. *N* is the number of the components. *a_in_*, *b_jn_* and *c_kn_* are elements of *A*, *B* and *C* matrices, respectively. The columns of *A* and *B* correspond to the pure excitation and emission spectra of fluorophores, respectively, and the column of *C* accounts for concentrations of each fluorophore in the *k* sample. *e_ijk_* is the residual error for the element *x_ijk_*. For PARAFAC algorithm, an alternating least squares approach was employed to solve the tri-linear component model, which minimized the sum of squares of the residuals *e_ijk_*.

In the present study, several PARAFAC models were performed to resolve the overlapped fluorescence signals in different mixtures. First, PARAFAC was performed on the four sets of mixtures separately, and the number of calibration and prediction samples used for PARAFAC analysis were 7 and 3 for each set, e.g. Model 1 was performed to decompose the data obtained from mixture 1, in which 1^#^–7^#^ samples were used as calibration samples and 8^#^–10^#^ as prediction samples. Analogously, Model 2--Model 4 were used to resolve the data in mixture 2–mixture 4, respectively. In addition, PARAFAC algorithm was performed on the four sets together, including 28 calibration samples and 12 predicted samples (PARAFAC Model 5), and a three-dimensional data array with a size of 61 × 151 × 40 was obtained. In the calibration step, C loadings are regressed against the real concentrations of each illegal drug in the mixtures to get a linear regression equation. In the prediction step, the obtained regression line can then be used to calculate the concentration of each illegal drug in the prediction samples.

## Results and discussion

### Individual standards

**Figure 2.**
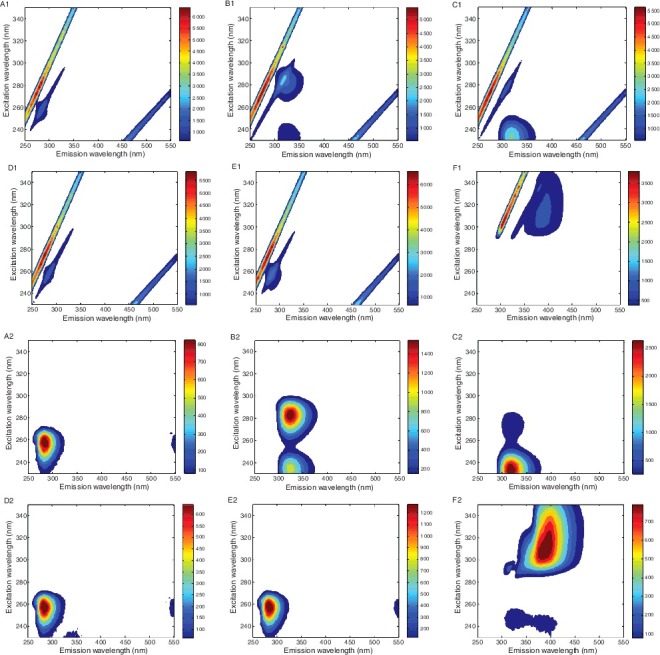
Excitation–emission matrix fluorescence spectrum of six ATS before (A1–F1) and after (A2–F2) handling the scattering signal. (A1, A2) MAM, (B1, B2) MDMA, (C1, C2) COC, (D1, D2) AM, (E1, E2) PSE and (F1, F2) CAF.

As illustrated in Figure 2(A1–F1), the signals of Rayleigh and Raman scattering are strong and cover the weak fluorescence signal of interest. These scattering singles do not contain any information concerning the fluorescence properties of the samples but lead the EEM array to deviate the tri-linear component model. Thus, it is necessary to pre-treat those measured spectra before analysing. In this study, the scattering signal was handled using interpolation in the areas affected by first- and second-order Rayleigh scattering and Raman scattering [21], and the results are shown in Figure 2(A2–F2).

ATS illegal drugs displayed a diversity of fluorescence characteristics. MAM exhibited a strong peak around λ_ex_/λ_em_ = 256/288 nm; MDMA was characterized by the presence of two peaks around λ_ex_/λ_em_ = 282/322 and 234/322 nm. COC also showed two peaks around λ_ex_/λ_em_ = 234/318 and 276/318 nm. CAF presented a very different characteristic, with a broad emission at 312/394 nm and another two weak peaks at 244/328 and 244/394 nm. Similar spectra were found among MAM, AM and PSE due to the minor structural differences of the three substances; one strong peak appeared at 256/288 nm for both AM and PSE.

To explore the linear range of each ATS, series of pure standards were prepared for each ATS individually. A significant positive linear relationship was observed between the concentrations and fluorescence intensity over the range 1.0–20.0, 0.01–0.1, 0.1–2.0, 2.0–20.0, 2.0–50.0 and 10.0–200 mg/L for MAM, MDMA, COC, AM, PSE and CAF, respectively.

### Determination of MAM, MDMA and its adulterants in mixtures separately for PARAFAC Model 1–Model 4

The number of components in the mixtures should be estimated before application of PARAFAC algorithm. The core consistency diagnostic (CORCONDIA) test was used to determine the number of components to avoid either overestimation or underestimation for PARAFAC model in the present work [22]. The appropriate number of components is attained when the core consistency drops to a lower value. Based on the core consistency test, two components were chosen for Model 1–Model 4 (Table 2), which were consistent with the actual components in ATS mixtures.

**Table 2.** Core consistency values for PARAFAC model (%).

Number of components	Model 1	Model 2	Model 3	Model 4	Model 5
1	100	100	100	100	100
2	100	100	100	100	99.9
3	0	0	0	0	99.8
4	0	0	0	0	98.9
5	0	0	0	0	26.3

**Figure 3.**
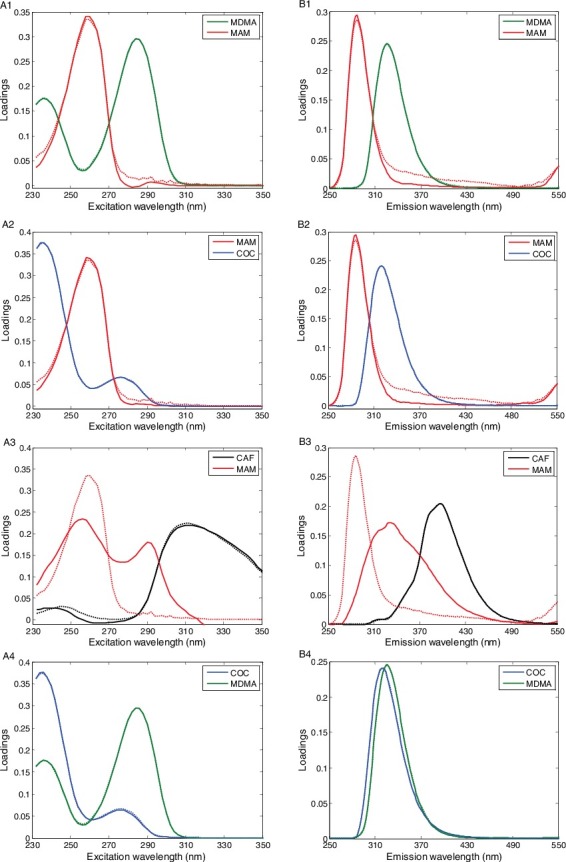
Loadings resolved from PARAFAC (solid line) and the actual spectra obtained from the individual compounds (dotted line). (A1–A4) excitation spectra and (B1–B4) emission spectra.

**Figure 4.**
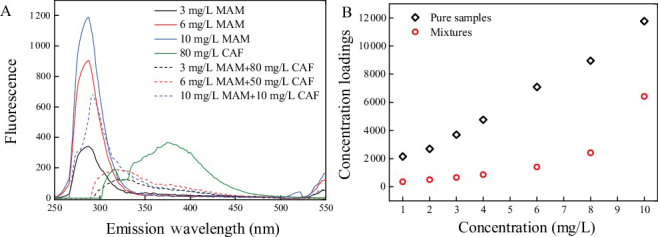
Emission spectra (A) and concentration loadings (B) of MAM in the presence of CAF.

Figure 3 shows the actual spectral profiles and loadings from decomposition of the EEM data array in each mixture with a factor number of 2. The dash line and solid line represent the actual and resolved profiles from PARAFAC model, respectively. The loading profiles were very similar to those actual profiles except for MAM in mixture 3 (MAM and CAF mixtures). Fluorescence quenching processes might be responsible for the bias of MAM loading profiles and decreased concentrations. When we added different amounts of CAF to pure MAM samples, the fluorescence intensity of MAM decreased; meanwhile, a new substance spectrum was observed (Figure 4) indicating that static quenching might be the major mechanism of the fluorescence quenching.

The prediction results in other three mixtures including mixture 1, mixture 2 and mixture 4 using PARAFAC algorithm are summarized in Table 3. Furthermore, figures of merit including sensitivity (SEN), selectivity (SEL), limit of determination (LOD), root-mean-square error of calibration (RMSEC) and prediction (RMSEP) and average recovery (AR) are also listed in Table 4. SEN, SEL and LOD were calculated as described by Olivieri [23,24] as

(2) $$\text{SEN}\;\text{=}\;\lambda {\left\{\left[{\left(A^{T}A\right)}^{\text{-1}}\right]*\left[\;{\left(B^{T}B\right)}^{\text{-1}}\right]\right\}}_{nn}^{\text{-1/2}}$$

(3) $$\text{SEL}\;\text{=}\;{\left\{\left[{\left(A^{T}A\right)}^{\text{-1}}\right]*\left[\;{\left(B^{T}B\right)}^{\text{-1}}\right]\right\}}_{nn}^{\text{-1/2}}$$

(4) LOD = 3.3 s(0)

where *nn* designates the (*n*, *n*) element of matrix [(*A^T^A*)^−1^]*[(*B^T^B*)^−1^], λ is the total signal for component *n* at unit concentration, and the symbol * indicates the Hadamard product. *A* and *B* correspond to the pure excitation and emission spectra of each ATS, *s*(0) is the standard error in the predicted concentration for the method blank samples. The accuracy of the model can also be estimated by RMSEC and RMSEP, which describes as

(5) $$\text{RMSEC}=\sqrt{\frac{\sum_{i=1}^{m}{\left(\overset{\wedge }{c_{i}}-c_{i}\right)}^{2}}{m}}$$

(6) $$\text{RMSEP}=\sqrt{\frac{\sum_{i=1}^{n}{\left(\overset{\wedge }{c_{i}}-c_{i}\right)}^{2}}{n}}$$

where *m* and *n* are the total number of components used in the calibration and prediction samples, respectively. ${\hat{C}}_{i}$is the predicted concentration in the *i*th sample and *C_i_* is the actual concentration in the *i*th sample.

**Table 3.** Predicted concentrations of MAM, MDMA and COC in different simulated mixtures (mg/L).

Mixture	Item	Predicted concentration		
1	No.	8^#^	9^#^	10^#^
	MAM	2.97	6.97	9.00
	MDMA	0.078	0.041	0.021
2	No.	18^#^	19^#^	20^#^
	MAM	1.98	4.99	7.98
	COC	0.89	0.60	0.30
4	No.	38^#^	39^#^	40^#^
	MDMA	0.030	0.049	0.079
	COC	0.79	0.60	0.30

**Table 4.** Figures of merit obtained from Model 1, Model 2 and Model 4.

	Mixture 1		Mixture 2		Mixture 4	
Statistic parameters	MAM	MDMA	MAM	COC	MDMA	COC
AR (%)	99.5 ± 0.5	102.4 ± 3.2	99.5 ± 0.5	99.6 ± 0.6	99.6 ± 0.8	100.1 ± 0.5
RMSEC (mg/L)	0.029	0.000 2	0.054	0.003	0.000 5	0.002
RMSEP (mg/L)	0.022	0.000 7	0.016	0.006	0.000 3	0.001
SEN (L/mg)	1 171.7	416 384	1 062.4	34 116	75 551	6 680
LOD (mg/L)	0.078	0.002 7	0.044	0.025	0.002	0.012

As shown in Table 4, the low RMSEC and RMSEP and high recovery value (close to 100%) indicated good performance of the proposed PARAFAC model for simultaneous determination of MAM, MDMA and COC.

### Determination of MAM, MDMA and its adulterants in mixtures simultaneously for PARAFAC Model 5

By comparing the results of simultaneous prediction MAM and MDMA in the presence of adulterants with those of individual prediction of MAM or MDMA in the presence of adulterants, we tried to discuss the potential of the PARAFAC model for determining the ATS illegal drugs in the presence of more interference. For PARAFAC Model 5, a three-dimensional data array with a size of 61 × 151 × 40 was obtained, including the mentioned mixture 1–mixture 4. The calibration sets were 1^#^–7^#^, 11^#^–17^#^, 21^#^–27^#^ and 31^#^–37^#^, and the remain samples were considered to be predicted samples.

**Figure 5.**
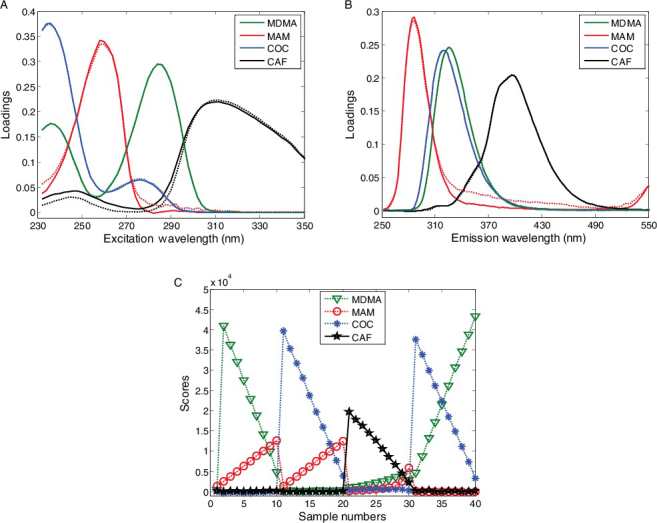
Actual and resolved profiles by PARAFAC Model 5. (A) Excitation profiles, (B) emission profiles and (C) concentration scores.

Just like predicted mixtures individually, the number of components was estimated prior to applying the PARAFAC algorithm. The result of CORCONDIA indicated that four factors were necessary, since the core consistency decreased lower than 40% when more factors were utilized. The loadings decomposed by PARAFAC model with four factors in excitation and emission modes are shown in Figure 5. The resolved excitation and emission profiles nicely matched those pure substances. However, it is obviously found that the loading scores of MAM in mixtures 21^#^–30^#^ were lower than any other values due to the fluorescence quenching caused by CAF. Therefore, in the calibration step, the sample numbers from 21^#^ to 27^#^ were excluded. Twenty-one resolved concentration scores were regressed against the real concentrations of MAM, MDMA and COC to get a linear calibration. The values of correlation coefficient (*r*) were all above 0.999, indicating that a good linear fit was obtained for each ATS in its calibration range.

**Figure 6.**
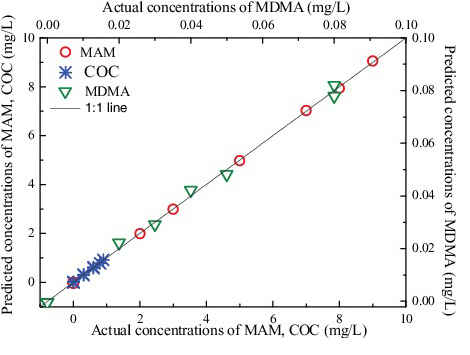
Predicted concentrations decomposed by PARAFAC Model 5 and actual concentrations for MAM, MDMA and COC.

Figure 6 presents the predicted concentrations decomposed by PARAFAC Model 5 against actual concentrations for MAM, MDMA and COC. Although the CAF was included in the PARAFAC Model 5, there was still a good agreement between the predicted and actual values for the MAM, MDMA and COC illegal drugs. PARAFAC algorithm has “second-order advantage”, which allows for quantitative analysis of interest in the presence of interferences.

The prediction results using PARAFAC Model 5 are summarized in Table 5. The average predicted recoveries were (99.8 ± 0.6)%, (101.6 ± 5.7)% and (99.8 ± 3.1)% for MAM, MDMA and COC, respectively, indicating that the PARAFAC algorithm was reliable for determining the mixtures. In addition, low errors in RMSEC and RMSEP implied good performance of the proposed PARAFAC model for determination of AM-type illegal drugs in the presence of adulterants. From the results of Table 5, we can conclude that MAM was the most selective compound in the mixture, while the selectivity of MDMA and COC was similar, in agreement with the spectra in Figure 5. The selectivity depends upon the characteristic of each illegal drug in the mixtures. However, the sensitivities are not in agreement with the selectivity. MDMA was more sensitive, yet MAM had the lowest sensitivity. The detection limit of this method was in the order of magnitude of 0.05 μg/mL.

**Table 5.** Figures of merit obtained from Model 5 using PARAFAC model.

Drugs	AR (%)	RMSEC (mg/L)	RMSEP (mg/L)	SEN (L/mg)	SEL	LOD (mg/L)	*t*_calculated_
MAM	99.8 ± 0.6	0.061	0.040	961.1	0.767	0.054	0.51
MDMA	101.6 ± 5.7	0.001 6	0.001 9	68 213	0.155	0.002 1	0.82
COC	99.8 ± 3.1	0.018	0.016	5 641	0.145	0.011	0.82

The paired *t*-test was used for determining the significance between the Model 1 and Model 4 including only one interference and the Model 5 containing up to three interferences [17]. For MAM, MDMA and COC, all the values of *t*_calculated_ were less than that of *t*_table_ = 2.57 at the 95% confidence level, indicating that the results obtained from different models had no significant difference at the 95% confidence level.

## Conclusion

In this study, a fast and reliable technique based on EEMs coupled with PARAFAC algorithm was proposed for determining the AM-type illegal drugs. The fluorescence characteristics of MAM, MDMA, COC, AM and PSE were investigated. Results showed that those illegal drugs displayed a diversity of fluorescence characteristics, with excitation wavelengths varying from 220 to 300 nm and emission wavelengths between 250 and 380 nm.

Five PARAFAC models were used to predict the content of MAM and MDMA in adulterated samples, including one to three interferences. Those models have no significant difference at the 95% confidence level. The average recoveries are all approximated to 100% for MAM and MDMA. In addition, the fluorescence method provides reliable results of AM-type illegal drugs quantification with high sensitivity, selectivity and low limit of detection according to the figures of merit. The proposed fast and reliable method based on the EEMs combined with PARAFAC algorithm will help law enforcement seize drug smuggling rapidly and then reduce the amount of the illegal drugs in the street market.

## Supplementary Material

supp_mat_figS1_TFSR.jpg
