# Supplementary figures and images for: Rapid and simple analysis of amphetamine-type illegal drugs using excitation–emission matrix fluorescence coupled with parallel factor analysis
Source: Forensic Sci Res. 2017 Jul 24;4(2):179–87. doi: 10.1080/20961790.2017.1349600 (PMC6610521; doi:10.1080/20961790.2017.1349600)

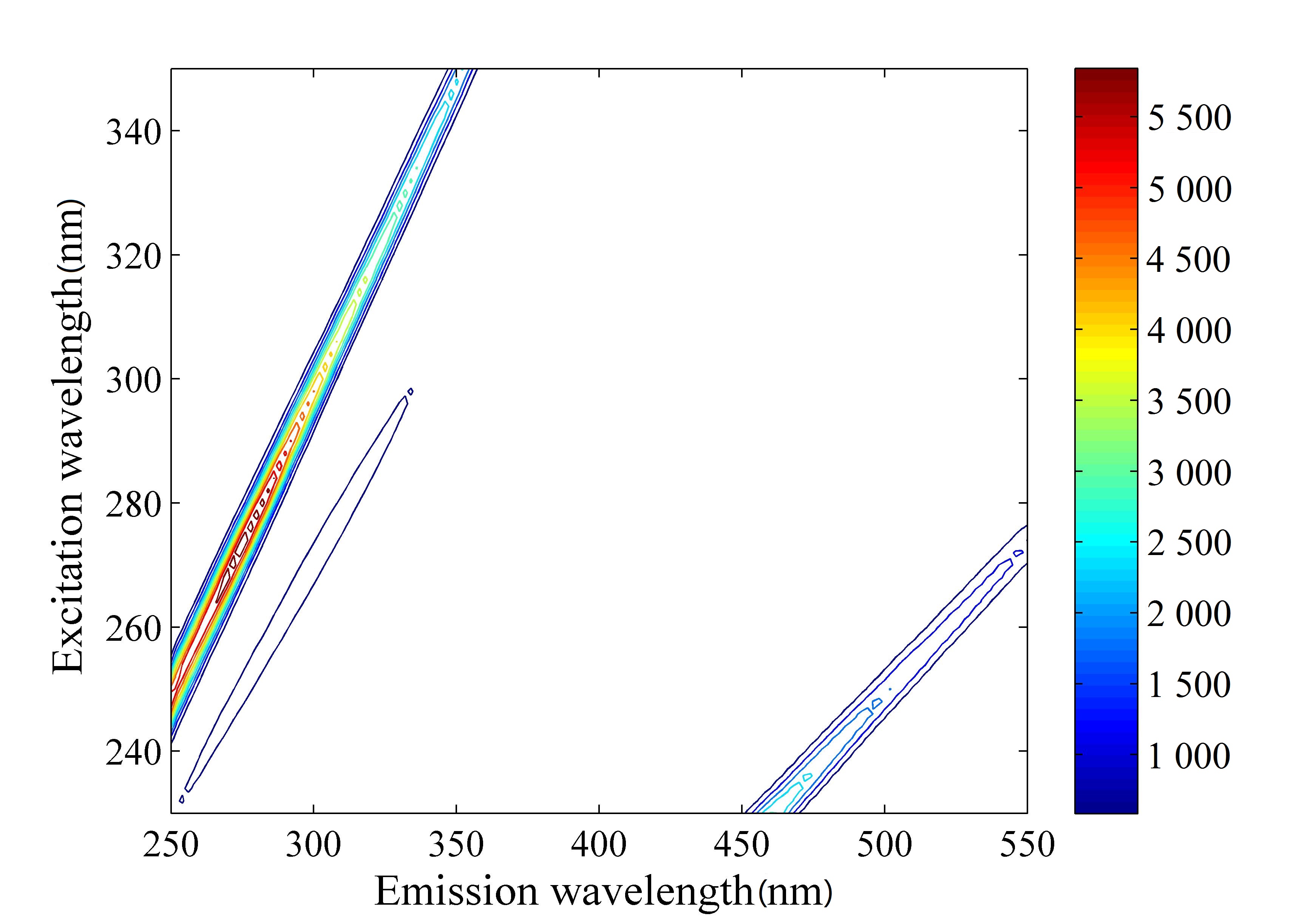

Supplement: supp_mat_figS1_TFSR.jpg [file TFSR_A_1349600_SM1410.jpg]
